# Symptom patterns before and after indolent systemic mastocytosis: A matched cohort analysis

**DOI:** 10.1016/j.jacig.2026.100687

**Published:** 2026-03-19

**Authors:** Kevin Y. Tse, Matt Zhou, Robert S. Zeiger, Chantal Avila, Mary Saparudin, Hiba Atif, Eric J. Puttock, Benjamin Lampson, Fagen Xie, Kerri Miller, Dakota Powell, Erin Sullivan, Chris Yuen, Wansu Chen

**Affiliations:** a Department of Allergy, Kaiser Permanente Medical Center, San Diego, Calif; b Department of Research and Evaluation, Kaiser Permanente Southern California, Pasadena, Calif; c Department of Clinical Science, Kaiser Permanente Bernard J. Tyson School of Medicine, San Diego, Calif; d Blueprint Medicines Corporation, Cambridge, Mass

**Keywords:** Systemic mastocytosis, symptoms, urticaria, osteoporosis, electronic health record

## Abstract

**Background:**

Systemic mastocytosis (SM) is a clonal mast cell disorder driven in most cases by *KIT* D816V mutation. The evolution of symptoms before and after diagnosis remains poorly understood.

**Objective:**

We sought to longitudinally describe various aspects of the real-world patient journey in the year leading up to and the year following diagnosis of SM.

**Methods:**

This retrospective, observational study included a previously confirmed cohort of patients with SM within a large integrated health system. We assessed physician-coded diagnoses, dispensing of medications, and patient-reported symptoms during the year before and after diagnosis, supplementing manual review with natural language processing outputs to enhance accuracy of symptom identification. Findings were compared with 2 matched comparison groups: patients with chronic spontaneous urticaria (CSU) and patients without SM or CSU.

**Results:**

Among 75 patients with SM (59 nonadvanced, 16 advanced), CSU patients with CSU, and 150 patients without SM or CSU, SM was associated with higher frequencies of cardiovascular disease, hepatosplenomegaly, osteoporosis, anemia, thrombocytopenia, eosinophilia, and elevated serum tryptase compared with both comparison groups. Gastrointestinal and neuropsychiatric symptoms were consistently more common in patients with SM, often persisting or worsening after diagnosis despite treatment. Advanced SM was distinguished by more pronounced hematologic and gastrointestinal abnormalities, whereas nonadvanced SM exhibited greater prevalence of cutaneous and neuropsychiatric symptoms.

**Conclusions:**

Patients with SM often present with heterogeneous, nonspecific symptoms that overlap with several other conditions, contributing to substantial diagnostic delays. Our findings underscore the need for more inclusive diagnostic approaches and improved management strategies that address the full spectrum of patient experience before and after diagnosis.

Systemic mastocytosis (SM) is a clonal mast cell disease, commonly driven by a somatic mutation in the *cKIT* gene. *KIT* gain-of-function mutations promote the accumulation of abnormal mast cells through sustained activation of *KIT* signaling pathways that enhance survival and support ligand-independent expansion within the bone marrow and other tissues. These mutations lead to uninhibited proliferation and an increased number of mast cells, resulting in accumulation and hyperactivation of abnormal mast cells in the bone marrow and various target organs.^1^, ^2^, ^3^ Clinically, SM manifests with a heterogeneous constellation of symptoms across multiple organ systems, reflecting both local mast cell infiltration and systemic mediator release.^4^

Despite increasing clinical awareness, SM remains underrecognized and misdiagnosed, particularly in patients presenting with non-specific symptoms. Estimated prevalence varies widely, ranging from 1 in 5,000 to 1 in 10,000 individuals.^5^, ^6^, ^7^, ^8^, ^9^ SM-related symptoms span cutaneous (eg, pruritus, urticaria pigmentosa, flushing), gastrointestinal (GI) (eg, bloating, abdominal pain, diarrhea), neuropsychiatric (eg, brain fog, anxiety, depression), musculoskeletal (eg, bone pain), and systemic (eg, fatigue, weight loss, anaphylaxis [which can be life-threatening]) domains. These manifestations may be episodic or chronic and are often misattributed to more common illnesses such as allergic diseases, functional GI syndromes, or mood disorders.^4^^,^^10^

Notably, many patients experience prolonged diagnostic delays, often cycling through multiple specialties over several years before receiving a correct diagnosis.^11^, ^12^, ^13^, ^14^ These delays arise not only from the unpredictable, multisystem nature of SM symptoms, but also from the fact that many of these symptoms are nonspecific and easily misattributed to more common conditions. For example, GI symptoms are frequently dismissed as minor or attributed to stress or anxiety rather than to SM.

Most existing literature has focused on symptoms at the time of diagnosis, and little is known about how symptoms evolve before diagnosis or how they change following diagnostic confirmation and treatment initiation. Understanding these temporal patterns may yield important diagnostic clues and support earlier recognition, particularly as new therapies targeting mast cell activity become available.

SM includes both advanced systemic mastocytosis (AdvSM), including aggressive SM and SM with associated hematologic neoplasm, and nonadvanced variants (non-AdvSM), such as indolent SM and smoldering SM. These subtypes differ markedly in disease course, symptom burden, and prognosis, and distinguishing between them is important for understanding the full clinical spectrum of SM and informing both clinical management and research. Despite the recognized symptom burden of SM, few studies have systematically examined the range and frequency of symptoms across the spectrum.

Our previous work identified a cohort of patients with physician-confirmed SM within the Kaiser Permanente Southern California (KPSC) health care system via retrospective electronic health record (EHR) chart review^11^ and a mail and online survey that revealed substantial unmet needs in symptom management and quality of life among patients with indolent SM.^12^ Many participants reported prolonged diagnostic delays. Building on these findings, the present study investigated the symptom trajectories in SM to characterize patterns of underrecognition before diagnosis and symptom persistence afterward. We compared symptom prevalence in the year before and after SM diagnosis with 2 matched groups: patients with chronic spontaneous urticaria (CSU) and patients without SM or CSU. Symptom patterns were also summarized descriptively for advanced and nonadvanced SM to explore differences across subtypes.

## Methods

### Study design and population

This retrospective, observational study was conducted within KPSC, a large integrated health care system. The study cohort included patients diagnosed with SM between 2008 and 2023, identified through manual chart abstraction of the KPSC EHR.^11^ Each patient was confirmed to meet the World Health Organization 2016 diagnostic criteria for SM,^15^^,^^16^ based on documented *KIT* D816V mutation and/or characteristic mast cell aggregates in bone marrow or other extracutaneous tissues.^11^ Eligible patients were adults (≥ 18 years old) with continuous enrollment in the KPSC health care system for at least 1 year before and 1 year after their SM diagnosis. This ensured complete capture of symptoms and other data elements during the specified time frames. The SM index date was defined as the first recorded diagnosis of SM within the system during the study period. Patient demographics, clinical characteristics, and pharmacy dispensing records were extracted from the KPSC research data warehouse.^17^ Physician notes (inpatient and outpatient) and electronic communications between patients and providers were extracted from Clarity, the data repository of the KPSC EHR system. Individual consent was waived under institutional review board approval.

To contextualize symptom patterns in patients with SM, we included 2 comparison groups identified within the same health care system. The first comprised patients with CSU (see Table E1 in this article’s Online Repository available at www.jaci-global.org), defined as having at least 1 CSU diagnosis by a KPSC allergist during the study period and no SM diagnosis. For patients with multiple CSU diagnoses, the first diagnosis was used as the index date. CSU was selected as a comparator because it represents a mast cell–mediated condition commonly managed by allergists, offering clinically meaningful context for mediator-related symptoms. The second comparison group consisted of patients without SM or CSU, serving as a baseline reference group. To minimize diagnostic overlap, we excluded individuals who ever developed SM or developed CSU within 5 years after their index date. Both comparison groups were matched to patients with SM based on age (±1 year), sex, and date of diagnosis (±1 year) at a 1:2:2 ratio and required continuous enrollment for 1 year before and after the index date. Patients diagnosed in 2008 and their matched counterparts were excluded from the symptom analysis because EHR data before that year were incompletely captured.

### Data collection

#### Demographics, comorbidities, laboratory findings, and medication exposures

We collected patient demographics (age, sex, race/ethnicity), comorbidities (eg, allergic and cardiovascular conditions), laboratory findings focusing on markers relevant to mast cell activation, hematologic abnormalities and systemic organ functions (eg, serum tryptase, blood counts, and liver function tests), and medication exposures targeting mast cell mediator release or treatments for allergic or systemic conditions (eg, epinephrine autoinjectors, antihistamines, corticosteroids), all assessed during the 1-year pre-diagnosis and 1-year post-diagnosis periods. Race and ethnicity were mainly self-reported according to standard US Census categories. The term Hispanic reflects the EHR ethnicity classification and does not refer to nationality or country of origin. The full list of data elements is presented in Table I, Table II, Table III, Table IV, and the definitions of the comorbidities are in Table E2 (in the Online Repository at www.jaci-global.org).

**Table I:** Demographics and lifestyle characteristics of SM, CSU, and non-SM/non-CSU cohorts

Characteristics	SM (n = 75)	CSU (n = 150)	Non-SM/non-CSU (n = 150)
Age (y)			
Mean (SD)	58.7 (13.7)	59.0 (13.4)	58.9 (13.6)
Median (IQR)	58 (47-68)	59 (48-69)	59 (48-69)
Range	33-88	33-85	32-88
Sex, no. (%)			
Male	41 (54.7)	82 (54.7)	82 (54.7)
Female	34 (45.3)	68 (45.3)	68 (45.3)
Race/ethnicity, no. (%)			
Non-Hispanic White	41 (54.7)	41 (27.3)	70 (46.7)
Hispanic	23 (30.7)	60 (40.0)	54 (36.0)
Black	6 (8.0)	14 (9.3)	10 (6.7)
Asian/Pacific Islander	4 (5.3)	33 (22.0)	15 (10.0)
Multiple/other/unknown	1 (1.3)	2 (1.4)	1 (0.7)
Length of health plan enrollment (y), mean (SD)	15.9 (15.5)	15.9 (11.0)	16.5 (12.0)
BMI (kg/m^2^)			
Mean (SD)	28.3 (5.5)	29.8 (6.8)	29.6 (6.1)
Unknown, no. (%)	2 (2.7)	2 (1.3)	25 (16.7)
Known, no. (%)	73 (97.3)	148 (98.7)	125 (83.3)
Underweight/normal	23 (31.5)	33 (22.3)	28 (22.4)
Overweight	28 (38.4)	56 (37.8)	50 (40.0)
Obese	22 (30.1)	59 (39.9)	47 (37.6)
Exercise (min/wk), no. (%)			
Unknown	17 (22.7)	23 (15.3)	62 (41.3)
Known	58 (77.3)	127 (84.7)	88 (58.7)
Did not exercise	18 (31.0)	56 (44.1)	33 (37.5)
Did exercise	40 (69.0)	71 (55.9)	55 (62.5)
Mean (SD)	147.2 (157.2)	116.5 (159.5)	137.8 (157.9)
Median (IQR)	90 (0-240)	70 (0-180)	120 (0-210)
Smoking status, no. (%)			
Unknown	6 (8.0)	2 (1.3)	28 (18.7)
Known	69 (92.0)	148 (98.7)	122 (81.3)
Yes	2 (2.9)	6 (4.1)	7 (5.7)
Never	45 (65.2)	100 (67.6)	79 (64.8)
Quit	22 (31.9)	41 (27.6)	36 (29.5)
Passive	0 (0)	1 (0.7)	0 (0)
Insurance types (mutually inclusive), no. (%)			
Commercial	44 (58.7)	90 (60.0)	94 (62.7)
Medi-Cal/other state programs	6 (8.0)	8 (5.3)	8 (5.3)
Medicare	25 (33.3)	46 (30.7)	48 (32.0)
Private pay	19 (25.3)	36 (24.0)	32 (21.3)

*BMI*, Body mass index; *IQR*, interquartile range.

**Table II:** Patient comorbidities 1 year before and 1 year after index date in SM, CSU, and non-SM/non-CSU cohorts

Comorbidities	SM (n = 75)		CSU (n = 150)		Non-SM/non-CSU (n = 150)	
	1 y before	1 y after	1 y before	1 y after	1 y before	1 y after
Diabetes	16 (21.3)	16 (21.3)	27 (18.0)	29 (19.3)	23 (15.3)	27 (18.0)
Hypertension	26 (34.7)	30 (40.0)	69 (46.0)	72 (48.0)	47 (31.3)	67 (44.7)
Malignancy	6 (8.0)	5 (6.7)	9 (6.0)	14 (9.3)	7 (4.7)	8 (5.3)
Hyperlipidemia	28 (37.3)	28 (37.3)	63 (42.0)	71 (47.3)	48 (32.0)	64 (42.7)
COPD	3 (4.0)	2 (2.7)	7 (4.7)	7 (4.7)	1 (0.7)	5 (3.3)
Cardiovascular disease	8 (10.7)	11 (14.7)	11 (7.3)	12 (8.0)	11 (7.3)	16 (10.7)
Coronary artery disease	5 (6.7)	5 (6.7)	8 (5.3)	9 (6.0)	8 (5.3)	14 (9.3)
Myocardial infarction	1 (1.3)	1 (1.3)	0 (0)	0 (0)	2 (1.3)	2 (1.3)
Coronary heart failure	2 (2.7)	6 (8.0)	2 (1.3)	4 (2.7)	1 (0.7)	2 (1.3)
Peripheral vascular disease	1 (1.3)	2 (2.7)	1 (0.7)	1 (0.7)	3 (2.0)	3 (2.0)
Connective tissue disease	9 (12.0)	7 (9.3)	12 (8.0)	13 (8.7)	12 (8.0)	15 (10.0)
Ulcer disease	0 (0)	1 (1.3)	2 (1.3)	2 (1.3)	1 (0.7)	1 (0.7)
Urticaria	1 (1.3)	4 (5.3)	39 (26.0)	150 (100)	0 (0)	1 (0.7)
Liver disease	7 (9.3)	8 (10.7)	4 (2.7)	7 (4.7)	4 (2.7)	5 (3.3)
Dysautonomia	0 (0)	0 (0)	0 (0)	0 (0)	0 (0)	0 (0)
IBS	3 (4.0)	5 (6.7)	3 (2.0)	1 (0.7)	2 (1.3)	3 (2.0)
IBD	2 (2.7)	6 (8.0)	2 (1.3)	3 (2.0)	4 (2.7)	5 (3.3)
Asthma	8 (10.7)	8 (10.7)	18 (12.0)	20 (13.3)	7 (4.7)	12 (8.0)
Allergic rhinitis	2 (2.7)	5 (6.7)	27 (18.0)	35 (23.3)	11 (7.3)	10 (6.7)
Atopic dermatitis	0 (0)	0 (0)	7 (4.7)	5 (3.3)	0 (0)	0 (0)
Anxiety	15 (20.0)	13 (17.3)	18 (12.0)	26 (17.3)	19 (12.7)	17 (11.3)
Depression	11 (14.7)	14 (18.7)	17 (11.3)	22 (14.7)	14 (9.3)	20 (13.3)
Dementia	2 (2.7)	4 (5.3)	3 (2.0)	6 (4.0)	6 (4.0)	4 (2.7)
Migraine	2 (2.7)	7 (9.3)	7 (4.7)	10 (6.7)	2 (1.3)	5 (3.3)
Cognitive impairment	1 (1.3)	1 (1.3)	4 (2.7)	2 (1.3)	1 (0.7)	2 (1.3)
Osteoporosis or osteopenia	10 (13.3)	18 (24.0)	13 (8.7)	17 (11.3)	8 (5.3)	14 (9.3)
Osteoarthritis	15 (20.0)	11 (14.7)	20 (13.3)	27 (18.0)	14 (9.3)	28 (18.7)
Fracture	8 (10.7)	8 (10.7)	5 (3.3)	8 (5.3)	0 (0)	6 (4.0)
Anaphylaxis	8 (10.7)	7 (9.3)	44 (29.3)	52 (34.7)	3 (2.0)	2 (1.3)
Hepatosplenomegaly						
Hepatomegaly	2 (2.7)	0 (0)	1 (0.7)	1 (0.7)	1 (0.7)	1 (0.7)
Splenomegaly	4 (5.3)	4 (5.3)	0 (0)	1 (0.7)	0 (0)	0 (0)
Lymphadenopathy	3 (4.0)	2 (2.7)	2 (1.3)	4 (2.7)	0 (0)	1 (0.7)
GERD	11 (14.7)	11 (14.7)	20 (13.3)	33 (22.0)	20 (13.3)	25 (16.7)
Hypotension	0 (0)	2 (2.7)	2 (1.3)	3 (2.0)	0 (0)	2 (1.3)

All values are no. (%). See Table E2 for *International Classification of Diseases, Ninth Revision* and *Tenth Revision* codes for comorbidities.

*COPD*, Chronic obstructive pulmonary disease; *GERD*, gastroesophageal reflux disease; *IBD*, inflammatory bowel disease; *IBS*, inflammatory bowel syndrome.

**Table III:** Patient laboratory characteristics 1 year before and 1 year after index date in SM, CSU, and non-SM/non-CSU cohorts

Characteristics	SM (n = 75)				CSU (n = 150)				Non-SM/non-CSU (n = 150)			
	1 y before		1 y after		1 y before		1 y after		1 y before		1 y after	
	No.	No. positive (%)	No.	No. positive (%)	No.	No. positive (%)	No.	No. positive (%)	No.	No. positive (%)	No.	No. positive (%)
Anemia (Hb <10 g/dL): Min	69	6 (8.7)	70	10 (14.3)	114	1 (0.9)	118	2 (1.7)	74	0 (0)	77	0 (0)
Leukopenia (<3.5 × 10^9^/L): Min	69	9 (13.0)	69	10 (14.5)	114	2 (1.8)	116	1 (0.9)	70	5 (7.1)	73	3 (4.1)
Platelets <100 × 10^9^/L: Min	69	5 (7.2)	69	9 (13.0)	114	3 (2.6)	116	2 (1.7)	70	1 (1.4)	73	1 (1.4)
Eosinophilia (>0.5 × 10^9^/L): Max	62	4 (6.5)	64	5 (7.8)	59	2 (3.4)	73	1 (1.4)	28	1 (3.6)	30	1 (3.3)
Monocytosis (>1.0 × 10^9^/L): Max	63	11 (17.5)	64	12 (18.8)	60	1 (1.7)	75	4 (5.3)	28	3 (10.7)	31	5 (16.1)
Serum tryptase >20 μg/L: Max	52	41 (78.8)	59	45 (76.3)	4	1 (25.0)	6	0 (0)	0	0 (0)	0	0 (0)
Serum tryptase >200 μg/L: Max	52	2 (3.8)	59	6 (10.2)	4	0 (0)	6	0 (0)	0	0 (0)	0	0 (0)
Serum LDH >240 IU/L: Max	19	0 (0)	27	2 (7.4)	7	0 (0)	6	1 (16.7)	0	0 (0)	2	1 (50.0)
SAP >140 IU/L: Max	54	5 (9.3)	60	10 (16.7)	36	0 (0)	54	2 (3.7)	18	1 (5.6)	22	0 (0)
β_2_-microglobulin >2.5 mg/L: Max	2	1 (50.0)	7	1 (14.3)	0	0 (0)	0	0 (0)	0	0 (0)	0	0 (0)
Serum cholesterol <120 mg/dL: Min	55	10 (18.2)	36	9 (25.0)	100	9 (9.0)	87	9 (10.3)	82	6 (7.3)	89	4 (4.5)
Serum vitamin B_12_ >1500 pg/mL: Max	19	1 (5.3)	16	1 (6.3)	16	0 (0)	19	0 (0)	11	0 (0)	9	0 (0)
Serum ferritin >200 ng/mL: Max	14	8 (57.1)	18	5 (27.8)	16	3 (18.8)	15	3 (20.0)	12	5 (41.7)	10	5 (50.0)
Serum ferritin <10 ng/mL: Min	14	0 (0)	18	0 (0)	16	1 (6.3)	15	0 (0)	12	0 (0)	10	0 (0)
ALT >2× upper limit: Max	66	1 (1.5)	65	1 (1.5)	102	0 (0)	103	0 (0)	69	1 (1.4)	83	0 (0)
AST >2× upper limit: Max	34	1 (2.9)	33	1 (3.0)	21	0 (0)	30	0 (0)	14	0 (0)	17	1 (5.9)
CRP ≥7.5 mg/L: Max	5	1 (20.0)	0	0 (0)	5	3 (60.0)	6	2 (33.3)	4	0 (0)	5	1 (20.0)
ESR ≥15 mm/h: Max	16	5 (31.3)	12	3 (25.0)	34	6 (17.6)	39	8 (20.5)	11	6 (54.5)	14	6 (42.9)

*ALT*, Alanine transaminase; *AST*, aspartate aminotransferase; *CRP*, C-reactive protein; *ESR*, erythrocyte sedimentation rate; *LDH*, lactate dehydrogenase; *Min*, minimum value within each time window; *Max*, maximum value within each time window; *SAP*, serum alkaline phosphatase.

**Table IV:** Medication dispensing or administration in 1 year before and 1 year after index date in SM, CSU, and non-SM/non-CSU cohorts

Dispensing medication	SM (n = 75)		CSU (n = 150)		Non-SM/non-CSU (n = 150)	
	1 y before	1 y after	1 y before	1 y after	1 y before	1 y after
Epinephrine autoinjector	17 (22.7)	27 (36.0)	19 (12.7)	34 (22.7)	0 (0)	0 (0)
Epinephrine injection	1 (1.3)	4 (5.3)	11 (7.3)	9 (6.0)	4 (2.7)	2 (1.3)
Systemic steroids	16 (21.3)	22 (29.3)	82 (54.7)	99 (66.0)	15 (10.0)	34 (22.7)
Inhaled corticosteroid	5 (6.7)	6 (8.0)	21 (14.0)	19 (12.7)	2 (1.3)	10 (6.7)
H_1_ antihistamines	16 (21.3)	19 (25.3)	48 (32.0)	59 (39.3)	2 (1.3)	11 (7.3)
H_2_ antihistamines	17 (22.7)	26 (34.7)	38 (25.3)	54 (36.0)	11 (7.3)	11 (7.3)
LTRAs and 5-LO inhibitors	4 (5.3)	7 (9.3)	12 (8.0)	28 (18.7)	2 (1.3)	4 (2.7)
Cromolyn sodium	5 (6.7)	11 (14.7)	0 (0)	0 (0)	0 (0)	0 (0)
Chemotherapy or targeted agents for SM	0 (0)	14 (18.7)	1 (0.7)	1 (0.7)	0 (0)	0 (0)
Osteoporosis drug	6 (8.0)	8 (10.7)	7 (4.7)	7 (4.7)	3 (2.0)	6 (4.0)
Biologics	0 (0)	0 (0)	1 (0.7)	16 (10.7)	0 (0)	0 (0)
PPI	13 (17.3)	15 (20.0)	25 (16.7)	27 (18.0)	21 (14.0)	28 (18.7)
Disease-modifying antirheumatic drugs (cyclosporine, methotrexate)	0 (0)	0 (0)	3 (2.0)	3 (2.0)	2 (1.3)	2 (1.3)

All values are no. (%).

*5-LO*, 5-Lipoxygenase; *LTRA*, leukotriene receptor antagonist; *PPI*, proton pump inhibitor.

#### Symptoms

A total of 23 symptoms were examined and grouped into the following 6 categories: cutaneous symptoms, GI symptoms, neuropsychiatric symptoms, musculoskeletal symptoms, systemic symptoms, and severe allergic reactions. A comprehensive list of symptom keywords and phrases was developed using clinical expertise, prior literature, and our previous patient survey (Table E3 in the Online Repository at www.jaci-global.org). Severe allergic reactions were defined using terms indicative of systemic or life-threatening responses, including anaphylaxis (Table E3). Symptoms associated with exclusionary conditions described in Table E4 (in the Online Repository at www.jaci-global.org) were not considered.

Two trained reviewers (H.A. and M.S.) conducted retrospective chart review of all available clinical notes and patient/provider communications (referred to as notes) for all eligible subjects flagged for the symptoms during the 1-year pre- and post-index periods. Newly identified keywords/phrases were added during review. To enhance accuracy, manual review was supplemented with outputs from a previously developed natural language processing (NLP) algorithm.^18^ Discrepancies between manual review and NLP results were adjudicated manually, and the confirmed classification was used for analysis.

**Fig 1:**
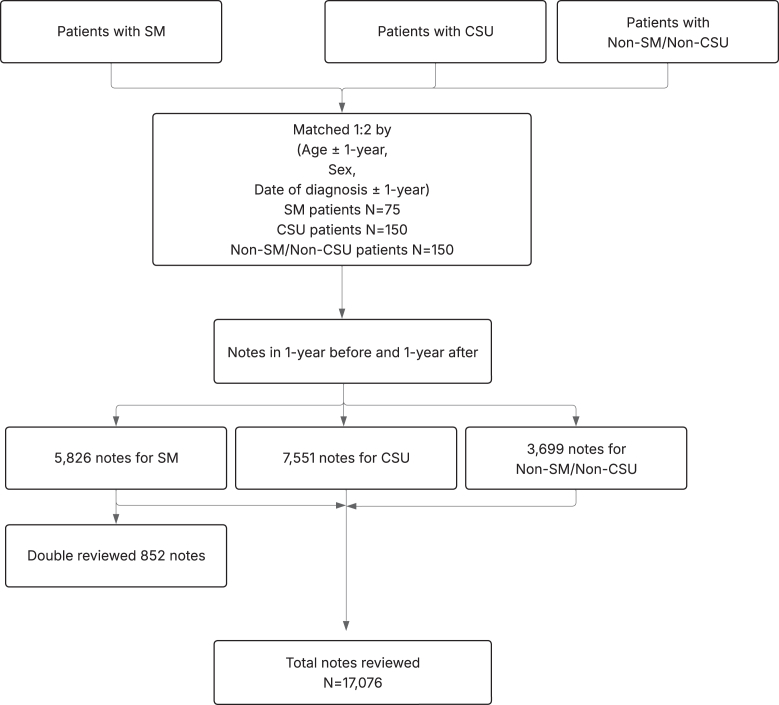
Flow of cohort selection and note review: 75 patients in SM cohort were matched 1:2 to 150 patients in CSU cohort and 150 patients in non-SM/non-CSU cohort, with a total of 17,076 notes reviewed (852 double-reviewed for reliability).

A total of 17,076 notes were reviewed, including 5,826 notes from patients with SM, 7,551 from patients with CSU, and 3,699 from non-SM/non-CSU patients (Fig 1). To ensure accuracy, the same 2 reviewers independently reviewed 14.6% of SM patient notes (n = 852). They verified whether each symptom truly occurred within the relevant time frame for each patient, using clinical context when necessary. Interrater reliability^19^ between the 2 reviewers was very high; most symptom categories achieved a Cohen’s κ value of 0.8 or greater, indicating strong agreement (Table E5 in the Online Repository at www.jaci-global.org). Because κ values could not be calculated for a few symptoms, we also included accuracy, which was defined as the number of matched records divided by the number of total records, and Gwet’s AC1.^20^ Any discrepancies in classification were resolved through consensus discussion, and the final agreed-on symptom data were used for analysis.

Symptom data were initially collected at the note level, with each mention documented along with its context. Subsequently, the data were collapsed to the patient level to summarize the presence or absence of symptoms for each patient during the pre- and post-diagnosis periods. We reported the combined prevalence for each symptom category at the patient level.

### Data analysis

Descriptive statistics summarized patient demographics, symptom prevalence, comorbidities, laboratory findings, and medication use across the SM and comparison groups. We also examined changes within each group from the year before to the year after diagnosis to evaluate the impact of diagnosis and subsequent management. Symptom patterns were summarized separately for patients with a non-AdvSM diagnosis (indolent SM or smoldering SM) and an AdvSM diagnosis (aggressive SM with associated hematologic neoplasm, aggressive SM, and mast cell leukemia) to explore potential differences across disease subtypes. Given the small sample size, no hypothesis testing was performed.

We also examined which clinical departments documented symptoms, assessed at the symptom category level (eg, cutaneous, GI, neuropsychiatric). For each category, we identified documentation by dermatology, allergy, gastroenterology, hematology/oncology, neurology/psychiatry, and internal medicine/family practice/urgent care. Results are presented separately for SM, CSU, and non-SM/non-CSU patients during the 1-year pre- and post-index periods.

## Results

Inclusion criteria were met by 75 patients with SM, 150 with CSU, and 150 with non-SM/non-CSU diagnoses. In the SM cohort, 59 patients were diagnosed with non-AdvSM, and 16 were diagnosed with AdvSM.

### Demographics

The SM cohort had a slightly higher proportion of male patients overall (Table I), driven by a male predominance in the AdvSM subgroup (Table E6 in the Online Repository at www.jaci-global.org). In contrast, the non-AdvSM subgroup showed a nearly even sex distribution. Of patients with SM, 30.7% identified as Hispanic, a proportion comparable to the comparison groups, with no major differences in overall racial or ethnic comparisons observed across cohorts (Table I). This Hispanic predominance was more marked in the AdvSM subgroup (Table E6).

### Comorbidities

Cardiovascular and liver diseases were more frequently diagnosed in patients with SM during the 1-year post-diagnosis period compared with both comparison groups (Table II). Urticaria was diagnosed in 1.3% of patients with SM before diagnosis and 5.3% after diagnosis. Neuropsychiatric conditions, including anxiety and depression, were more prevalent in patients with SM before diagnosis. Osteoporosis or osteopenia was also more frequently reported in patients with SM both before and after diagnosis. Anaphylaxis was more often documented in the SM cohort than in the non-SM/non-CSU cohort, though frequency of anaphylaxis in the CSU cohort was even higher. Hepatomegaly, splenomegaly, and lymphadenopathy were likewise more frequent in the SM cohort (Table II), particularly in the AdvSM subgroup (Table E7 in the Online Repository at www.jaci-global.org).

By SM subtype (Table E7), AdvSM patients showed higher frequencies of hypertension, cardiovascular disease, and liver disease in both time periods. Non-AdvSM patients exhibited higher frequencies of asthma, allergic rhinitis, and migraines both before and after the index date, whereas gastroesophageal reflux disease was more prevalent in these patients before the index date only. Osteoporosis or osteopenia was more prevalent in non-AdvSM patients before diagnosis, whereas it was increased in AdvSM patients after diagnosis.

### Laboratory findings

Distinct hematologic and metabolic abnormalities were more frequently reported in the SM cohort than in the 2 comparison groups (Table III), including higher frequencies of anemia, thrombocytopenia, leukopenia, monocytosis, and elevated serum tryptase. Elevated serum alkaline phosphatase and low total cholesterol were also more frequent. Eosinophilia, though not traditionally emphasized in SM, was overrepresented among patients with SM before diagnosis. Compared with non-AdvSM patients, AdvSM patients had a higher prevalence of all of these hematologic and metabolic abnormalities (Table E8 in the Online Repository at www.jaci-global.org).

### Medication dispensing

Medication dispensing patterns reflected differential management strategies across groups (Table IV). Epinephrine autoinjectors were dispensed more frequently in patients with SM than in the other groups. In contrast, systemic and inhaled corticosteroids and antihistamines (both H_1_ and H_2_) were dispensed more frequently to patients with CSU in the year before diagnosis compared with SM and non-SM/non-CSU groups. Leukotriene receptor antagonists and 5-lipoxygenase inhibitors were prescribed most frequently in the CSU cohort, with minimal use in the SM cohort. However, cromolyn sodium, in various formulations (eg, oral, intranasal, eyedrop), was dispensed almost exclusively to patients with SM both before and after date of diagnosis. Osteoporosis treatments were also most frequently dispensed in the SM group, consistent with reported bone-related comorbidity. Chemotherapeutics or targeted SM agents were used almost exclusively in patients with SM and mostly after diagnosis.

In SM subtypes (Table E9 in the Online Repository at www.jaci-global.org), antihistamines were dispensed more frequently in non-AdvSM patients. Leukotriene receptor antagonists and 5-lipoxygenase inhibitors were exclusive to this group. Chemotherapy or targeted SM agents use increased after diagnosis in AdvSM patients.

### Symptom burden

#### Cutaneous symptoms

**Fig 2:**
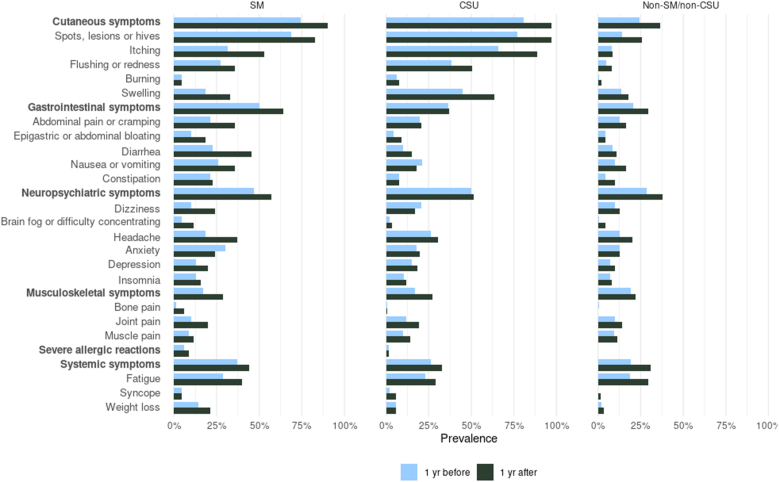
Symptom prevalence 1 year before and after 1 year index date in SM, CSU, and non-SM/non-CSU cohorts.

Patients with SM reported higher frequencies of cutaneous symptoms compared with the non-SM/non-CSU group, but reported them less often than patients with CSU (Fig 2; Table E10 in the Online Repository at www.jaci-global.org). Hives and rashes were most frequently reported in the SM group, followed by flushing or redness and itching. Swelling was less common in the SM group than the CSU group. Across all 3 groups, cutaneous symptoms increased after diagnosis.

Interestingly, non-AdvSM patients reported more cutaneous symptoms overall than AdvSM patients (Table E11), particularly spots/lesions/hives, itching, and flushing/redness. In contrast, burning and swelling were more commonly reported in AdvSM.

#### GI symptoms

GI symptoms were more prevalent in patients with SM both before and after diagnosis relative to the 2 comparison groups (Fig 2; Table E10). Abdominal pain, bloating, diarrhea, and nausea/vomiting increased after SM diagnosis. These trends were observed in both non-AdvSM and AdvSM subgroups, but were especially pronounced in the AdvSM subgroup after diagnosis (Table E11).

#### Neuropsychiatric symptoms

Brain fog, anxiety, and insomnia were reported more frequently in patients with SM than in the 2 comparison groups both before and after diagnosis (Fig 2; Table E10). Compared with AdvSM patients, non-AdvSM patients more often reported a range of neuropsychiatric symptoms, including dizziness, brain fog, headache, anxiety, and depression (Table E11).

#### Musculoskeletal symptoms

Overall, musculoskeletal symptoms were reported more frequently after diagnosis in all 3 groups (Fig 2; Table E10). Joint pain was most common, followed by muscle pain. AdvSM patients exhibited more musculoskeletal symptoms, particularly joint pains and muscle pains (Table E11), compared with non-AdvSM patients.

#### Systemic symptoms and severe allergic reactions

Fatigue and weight loss occurred more often in patients with SM across both time periods than in comparison groups (Fig 2; Table E10). Severe allergic reactions, including anaphylaxis, were also more common in SM. These symptoms were primarily reported by non-AdvSM patients; AdvSM patients had no documented severe allergic reactions (Table E11).

#### Symptoms documented by department

In all 3 groups, primary care physicians (internal medicine, family practice, or urgent care) documented the majority of symptoms (Table V). In patients with SM, documentation by specialists (especially hematology/oncology, allergy, and gastroenterology) increased substantially after diagnosis. GI symptoms, in particular, were more frequently documented by specialties after diagnosis, an observation not mirrored in the CSU group or non-SM/non-CSU group.

**Table V:** Prevalence of symptom documentation across clinical specialties in the 1 year before and after index date among SM, CSU, and non-SM/non-CSU patients

Symptom category	Provider type	SM (n = 70)		CSU (n = 140)		Non-SM/non-CSU (n = 140)	
		1 y before	1 y after	1 y before	1 y after	1 y before	1 y after
Cutaneous	Dermatology	37 (52.9)	22 (31.4)	27 (19.3)	40 (28.6)	9 (6.4)	13 (9.3)
	Allergy	10 (14.3)	22 (31.4)	29 (20.7)	116 (82.9)	0 (0)	0 (0)
	Gastroenterology	0 (0)	2 (2.9)	9 (6.4)	1 (0.7)	0 (0)	1 (0.7)
	Hem-onc	20 (28.6)	52 (74.3)	3 (2.1)	6 (4.3)	1 (0.7)	2 (1.4)
	Neurology/psychiatry	1 (1.4)	4 (5.7)	0 (0)	6 (4.3)	1 (0.7)	2 (1.4)
	IM/FP/UC	43 (61.4)	40 (57.1)	109 (77.9)	121 (86.4)	31 (22.1)	47 (33.6)
GI	Dermatology	4 (5.7)	2 (2.9)	1 (0.7)	0 (0)	0 (0)	0 (0)
	Allergy	5 (7.1)	15 (21.4)	0 (0)	4 (2.9)	1 (0.7)	1 (0.7)
	Gastroenterology	5 (7.1)	8 (11.4)	7 (5.0)	5 (3.6)	3 (2.1)	4 (2.9)
	Hem-onc	6 (8.6)	23 (32.9)	3 (2.1)	2 (1.4)	1 (0.7)	2 (1.4)
	Neurology/psychiatry	5 (7.1)	4 (5.7)	1 (0.7)	2 (1.4)	1 (0.7)	1 (0.7)
	IM/FP/UC	28 (40.0)	35 (50.0)	48 (34.3)	46 (32.9)	27 (19.3)	40 (28.6)
Neuropsychiatric	Dermatology	1 (1.4)	3 (4.3)	1 (0.7)	1 (0.7)	1 (0.7)	0 (0)
	Allergy	5 (7.1)	8 (11.4)	3 (2.1)	12 (8.6)	0 (0)	1 (0.7)
	Gastroenterology	2 (2.9)	2 (2.9)	1 (0.7)	0 (0)	1 (0.7)	1 (0.7)
	Hem-onc	0 (0)	9 (12.9)	1 (0.7)	1 (0.7)	0 (0)	0 (0)
	Neurology/psychiatry	12 (17.1)	13 (18.6)	19 (13.6)	21 (15)	11 (7.9)	14 (10.0)
	IM/FP/UC	27 (38.6)	37 (52.9)	65 (46.4)	62 (44.3)	36 (25.7)	50 (35.7)
Musculoskeletal	Dermatology	0 (0)	0 (0)	0 (0)	0 (0)	0 (0)	0 (0)
	Allergy	2 (2.9)	3 (4.3)	1 (0.7)	3 (2.1)	1 (0.7)	1 (0.7)
	Gastroenterology	1 (1.4)	1 (1.4)	1 (0.7)	1 (0.7)	1 (0.7)	0 (0)
	Hem-onc	2 (2.9)	3 (4.3)	0 (0)	0 (0)	0 (0)	1 (0.7)
	Neurology/psychiatry	0 (0)	2 (2.9)	2 (1.4)	0 (0)	0 (0)	0 (0)
	IM/FP/UC	10 (14.3)	17 (24.3)	22 (15.7)	35 (25.0)	26 (18.6)	30 (21.4)
Severe allergic reactions	Dermatology	1 (1.4)	0 (0)	0 (0)	0 (0)	0 (0)	0 (0)
	Allergy	1 (1.4)	3 (4.3)	0 (0)	0 (0)	0 (0)	0 (0)
	Gastroenterology	0 (0)	0 (0)	0 (0)	0 (0)	0 (0)	0 (0)
	Hem-onc	0 (0)	4 (5.7)	0 (0)	0 (0)	0 (0)	0 (0)
	Neurology/psychiatry	0 (0)	0 (0)	0 (0)	0 (0)	0 (0)	0 (0)
	IM/FP/UC	3 (4.3)	2 (2.9)	2 (1.4)	2 (1.4)	0 (0)	0 (0)
Systemic	Dermatology	2 (2.9)	1 (1.4)	0 (0)	0 (0)	0 (0)	0 (0)
	Allergy	3 (4.3)	5 (7.1)	1 (0.7)	5 (3.6)	1 (0.7)	1 (0.7)
	Gastroenterology	2 (2.9)	2 (2.9)	2 (1.4)	1 (0.7)	1 (0.7)	2 (1.4)
	Hem-onc	8 (11.4)	21 (30.0)	1 (0.7)	2 (1.4)	1 (0.7)	1 (0.7)
	Neurology/psychiatry	6 (8.6)	3 (4.3)	6 (4.3)	8 (5.7)	3 (2.1)	7 (5.0)
	IM/FP/UC	19 (27.1)	23 (32.9)	29 (20.7)	41 (29.3)	26 (18.6)	38 (27.1)

All values are no. (%).

*Hem-onc*, Hematology or oncology; *IM/FP/UC*, internal medicine/family practice/urgent care.

## Discussion

This study offers a longitudinal view into the symptom trajectories of patients with SM, using both structured and unstructured data to trace how symptoms emerge and evolve in the diagnostic process. By comparing the SM cohort with matched CSU and general cohorts, we sought to contextualize rather than contrast diseases. CSU serves as a reference for mast cell–mediated symptoms, and the general cohort provides a population baseline for nonspecific findings, helping interpret SM symptom trajectories over time.

Our findings reaffirm the multisystem nature of SM, encompassing cutaneous, GI, neuropsychiatric, musculoskeletal, and systemic domains.^10^^,^^12^^,^^21^^,^^22^ In contrast to prior studies that focused exclusively on symptoms at diagnosis, we examined how symptoms were documented in the year before and after diagnosis. The higher prevalence of symptoms documented after diagnosis likely reflects increased clinical attention, specialist involvement, and more comprehensive diagnostic coding once SM is recognized, rather than delayed recognition of symptom onset. Our intent was not to determine whether symptoms were previously overlooked or to estimate diagnostic delay, which would require patient-reported timelines and detailed clinical adjudication, but rather to characterize how symptoms appear in the routine EHR documentation. Notably, many symptoms persisted after diagnosis despite increased clinical attention and treatment.

Although cutaneous symptoms were common in patients with SM, they were reported more frequently in the CSU cohort, reinforcing that urticaria alone is not a distinguishing feature of SM. In contrast, GI symptoms were reported more commonly in the SM cohort than in either comparison group in both the year before and the year after diagnosis. Notably, GI symptoms were documented more frequently following SM diagnosis, despite increased clinical attention and pharmacologic intervention. This post-diagnosis increase likely reflects a combination of more systematic symptom elicitation and documentation once SM is recognized, persistence of symptoms that remain incompletely controlled with current therapies, progression over time, or a combination of these factors. Increased documentation of GI symptoms after diagnosis coincided with greater involvement of allergy, gastroenterology, and hematology/oncology clinicians (Table V). Despite their prominence in real-world clinical documentation, GI symptoms receive relatively limited emphasis in commonly used SM screening tools such as the REMA (Red Española de Mastocitosis [Spanish Network on Mastocytosis]) score^22^ and NICAS (NIH Idiopathic Clonal Anaphylaxis Score)^23^, which were developed to screen for SM in populations presenting with idiopathic anaphylaxis and therefore prioritize acute cutaneous, hemodynamic (syncope/presyncope), and laboratory features. These observations suggest that GI symptoms, particularly when occurring alongside other features suggestive of SM, may warrant greater consideration in future approaches to SM evaluation, while maintaining sufficient specificity to avoid overdiagnosis.

GI symptoms in SM have been widely attributed to mast cell infiltration of the GI tract and systemic mediator release. These processes may contribute to local inflammation; malabsorption; and functional disturbances such as abdominal pain, bloating, and diarrhea. Although our data do not allow direct assessment of these mechanisms, they provide context for the elevated prevalence and persistence of GI symptoms observed among patients with SM.

Persistent GI symptoms, especially when occurring alongside cutaneous findings typical of SM, may heighten clinical suspicion for underlying mast cell disease and prompt consideration of further diagnostic evaluation. However, our study was not designed to assess diagnostic performance or predictive value, and prospective studies are needed to determine whether such symptom combinations meaningfully inform screening or diagnostic strategies.

Musculoskeletal symptoms were reported more frequently among patients with SM, particularly patients with AdvSM. Although these findings are consistent with known skeletal involvement and systemic inflammation observed in SM, the modest differences observed and the nonspecific nature of these symptoms limit their interpretability. Accordingly, musculoskeletal symptoms should be viewed as part of the overall symptom burden rather than as indicators of disease-specific pathophysiology.

Because many symptoms observed in SM are also common in the general population, careful interpretation is warranted. Our findings are not intended to suggest that nonspecific symptoms such as depression, anxiety, or joint pain should independently trigger diagnostic evaluation for SM. Rather, these symptoms highlight the broad and often overlapping clinical burden experienced by patients with SM. Their inclusion reflects the heterogeneous nature of symptom presentation and the frequent coexistence of common and nonspecific symptoms in this population. Future studies will explore whether certain combinations of symptoms, such as GI symptoms with cutaneous or neuropsychiatric features, provide additional clinical insight.

While anaphylaxis was more commonly documented in the notes of patients with SM compared with the non-SM/non-CSU group (Fig 2; Table E10), it was most frequently coded by physicians in the CSU group (Table II). This discrepancy suggests several possibilities: Patients with SM may self-report anaphylaxis-like episodes that clinicians do not consistently interpret as anaphylaxis; clinicians may face diagnostic uncertainty or lack specific training in recognizing mast cell–mediated anaphylaxis; or anaphylaxis may be overdiagnosed or misclassified in CSU, consistent with prior reports.^23^ Variation across different clinical scoring systems may further contribute to inconsistent classification.

Medication dispensing patterns indicate a similar contrast across groups. Patients with CSU were more likely to receive antihistamines, corticosteroids, and leukotriene-modifying agents, reflecting a more established therapeutic algorithm. In contrast, patients with SM, despite experiencing multisystem symptoms that often persisted or even worsened after diagnosis, received relatively limited therapeutic options beyond SM-specific agents such as cromolyn sodium. Targeted SM therapeutics were used primarily in patients with AdvSM and largely after diagnosis, reflecting both the disease severity and the timing of therapeutic availability. Although these patterns may be institution specific and not fully generalizable, they highlight substantial variability in practice and ongoing treatment gaps. Together, these medication patterns suggest ongoing challenges in aligning symptom burden with available treatment strategies in routine SM care.

Our findings reaffirm and extend previous research documenting the systemic nature of SM, including its hematologic, immunologic, and metabolic manifestations.^1^^,^^3^ Observed patterns such as elevated tryptase and serum alkaline phosphatase levels, cytopenia, and eosinophilia are consistent with known disease pathways and underscore the broad multiorgan involvement characteristic of SM. Notably, though not traditionally emphasized in SM classification, eosinophilia was disproportionately represented in our cohort, particularly in the first year following diagnosis. This was especially notable in the AdvSM subgroup. This finding supports growing evidence that elevated eosinophils may indicate more severe or treatment-resistant SM.^24^

Recent efforts to harmonize SM classification across global consortia (World Health Organization, European Competence Network on Mastocytosis, American Initiative in Mast Cell Diseases, International Consensus Classification) have emphasized genetic and molecular criteria, including *KIT* D816V mutation status, CD30 expression, and bone marrow histopathology.^25^ Although these advances improve diagnostic precision, reliance on biomarkers with limited sensitivity may overlook clinically meaningful patient-reported symptoms and live experiences, potentially resulting in missed cases of SM. Diagnostic approaches may therefore need to evolve in parallel to better integrate longitudinal clinical features and patient-reported outcomes, while maintaining sufficient specificity to avoid overdiagnosis. Many common symptoms, such as depression, anxiety, fatigue, and musculoskeletal pain, are prevalent in the general population and, when present in isolation, are neither characteristic of mastocytosis nor indicative of a high index of suspicion.

This study has several strengths. To our knowledge, it is the first to integrate detailed manual chart review, including provider and patient communications, with quantitative analysis of laboratory, medication, and symptom data to map SM symptom trajectories across time. In addition, review findings were supplemented with outputs from an NLP algorithm specifically developed for SM symptom identification. The inclusion of 2 comparison groups allowed for contextual interpretation of symptom burden, helping to disentangle which features may be more specific to SM while hoping to avoid the risk of overdiagnosis based on the presence of common and nonspecific symptoms (eg, fatigue, musculoskeletal pain, anxiety).

Several limitations should be noted. This retrospective analysis relied on clinician documentation and is subject to underreporting, misclassification, and interpretive variability, which patient-provider communications could mitigate but not eliminate. Symptom coding inconsistencies, particularly for anaphylaxis, may have affected observed frequencies, especially among patients with CSU initially evaluated in emergency or urgent care settings. Post-diagnosis coding may also reflect incomplete integration of specialist documentation. In addition, hereditary alpha-tryptasemia testing was not routinely available, limiting assessment of its contribution to baseline tryptase levels, although all SM diagnoses met World Health Organization 2016 criteria. Finally, the modest SM sample size limited statistical inference and detailed subgroup analyses.

### Conclusion

This study highlights the epistemic and clinical gaps that continue to shape the diagnostic and therapeutic landscapes of SM. Our findings call for diagnostic paradigms that integrate patient-reported experiences, particularly GI symptoms, alongside molecular and histopathologic testing. The future of SM care lies in bridging these perspectives to reduce diagnostic delays and improve symptom management.

**Clinical implications**

Patients with SM present with various symptoms and comorbidities. Increasing awareness of these underrecognized findings may help physicians identify patients with SM earlier.

## Disclosure statement

Research reported in this publication was supported by a grant from Blueprint Medicines (Cambridge, Mass) to Kaiser Permanente Southern California. The content is solely the responsibility of the authors and does not necessarily represent the official views of the funding agency.

Disclosure of potential conflict of interest: R. S. Zeiger reports consulting fees from AstraZeneca, grant support from Sanofi, and royalties from UpToDate. B. Lampson, K. Miller, D. Powell, C. Yuen, and E. Sullivan are employed by Blueprint Medicines. The rest of the authors declare that they have no relevant conflicts of interest.
