# Supplementary material for: Symptom patterns before and after indolent systemic mastocytosis: A matched cohort analysis
Source: J Allergy Clin Immunol Glob. 2026 Mar 19;5(3):100687. doi: 10.1016/j.jacig.2026.100687 (PMC13091985; doi:10.1016/j.jacig.2026.100687)
Supplement: Supplementary Figs and Tables [file mmc1.docx]

**Online Supplemental Materials**

**Table E1. Chronic Spontaneous Urticaria cases.**

Chronic spontaneous urticaria (CSU) cases were defined using ICD-9 and ICD-10 codes, with the following additional criteria:

| Code version |  |
| --- | --- |
| ICD-10^a^ | (1) either 2 outpatient diagnoses of ICD-10 code L50.1 (idiopathic urticaria), L50.8 (other specified urticaria), or L50.9 (urticaria, unspecified) at least 6 weeks apart; or  (2) one outpatient diagnosis of L50.1, L50.8, or L.50.9 plus one diagnosis of T78.3 (angioneurotic edema) at least 6 weeks apart. |
| ICD-9^b^ | (1) either 2 outpatient diagnosis of ICD-9 code 708.1 (idiopathic urticaria), 708.8 (other specified urticaria), or 708.9 (urticaria, unspecified) at least 6 weeks apart; or  (2) one outpatient diagnosis of 708.1, 708.8, or 708.9 plus one diagnosis of 995.1 (angioneurotic edema) at least 6 weeks apart. |

Source of the algorithm:

^a^[Increased Risk of Chronic Spontaneous Urticaria in Patients With Autoimmune Thyroid Diseases: A Nationwide, Population-based Study - PubMed](https://pubmed.ncbi.nlm.nih.gov/28497925/)

^b^[Validation of an ICD-9-based claims algorithm for identifying patients with chronic idiopathic/spontaneous urticaria - PubMed](https://pubmed.ncbi.nlm.nih.gov/25771155/)

**Table E2. Comorbidities reported in Table 2 were defined by the following ICD-9 and ICD-10 codes.**

| **Comorbid condition** | **ICD-9** | **ICD-10** |
| --- | --- | --- |
| Diabetes | 250*, 357.2*, 362.0*, 366.41 | E08*, E09*, E10*, E11*, E12* |
| Hypertension | 401.0, 401.1, 401.9, 402.00, 402.01, 402.10, 402.11, 402.90, 402.91, 405.01, 405.09, 405.11, 405.19, 405.91, 405.99, 416.8 | I10, I11.0, I11.9, I15.0, I15.1, I15.2, I15.8, I15.9, I16.0, I16.1, I16.9, I27.20, I27.21, I27.22, I27.23, I27.24, I27.29, I27.89, N26.2 |
| Malignancy | 202.80- 202.88, 203.80, 203.81, 203.82, 209.30- 209.36, 140*, 141.0*- 141.6*, 141.8*, 141.9*, 142.0*, 142.1*, 142.2*, 142.8*, 142.9*, 143*, 144*, 145.0*- 145.6*, 145.8*, 145.9*, 146*, 147*, 148.0*- 148.3*, 148.8*, 148.9*, 149*- 153*, 154.0*- 154.3*, 154.8*, 155*, 156.0*- 156.2*, 156.8*, 156.9*, 157*, 158*, 159*, 160.0*- 160.5*, 160.8*, 160.9*, 161*, 162.0*- 162.5*, 162.8*, 162.9*, 163*, 164.0*- 164.3*, 164.8*, 164.9*, 165*, 170*, 171.0*, 171.2*- 171.9*, 172*, 173*, 174*, 175*, 176*, 179*, 180*, 181*, 182*, 183.0*, 183.2*, 183.3*, 183.4*, 183.5*, 183.8*, 183.9*, 184.0*- 184.4*, 184.8*, 184.9*, 185*, 186*, 187.1*- 187.9*, 188*, 189.0*- 189.4*, 189.8*, 189.9*, 190*, 191*, 192.0*- 192.3*, 192.8*, 192.9*, 193*, 194.0*, 194.1*, 194.3*- 194.6*, 194.8*, 194.9*, 195.0*- 195.5*, 195.8*, 196*, 197*, 198*, 199*, 200.0*- 200.8*, 201*, 202.0*, 202.1*, 202.2*, 202.4*, 202.7*, 203.0*, 203.1*, 204.2*, 204.8*, 204.9*, 209.0*, 209.1*, 209.2*, 209.7*, 230*, 231*, 232*, 233.0*- 233.7*, 233.9*, 234*, 273.3* | C00*- C26*, C30*- C34*, C37*- C41*, C43*- C58*, C4A*, C60*- C75*, C76.0*, C76.1*, C76.2*, C76.3*, C76.4*, C76.5*, C76.8*,C77*, C78*, C79*, C7A*, C7B*, C80*- C86*, C88*, C90*, C91.4*, C91.9*, C91.Z*, D00*- D07*, D09* |
| Hyperlipidemia | 272* | E78* |
| COPD | 491.0, 491.1, 491.20, 491.21, 491.22, 491.8, 491.9, 492.0, 492.8, 493.20, 493.21, 493.22, 496 | J41.0, J41.1, J41.8, J42, J43.0, J43.1, J43.2, J43.8, J43.9, J44.0, J44.1, J44.9 |
| Cardiovascular disease | Includes MI, CAD, CHF, PVD, see below | Includes MI, CAD, CHF, PVD, see below |
| MI part of coronary artery disease | 410*, 411.0, 412 | 121*, 122*, 123* |
| Coronary artery disease | 410*, 411*, 412*, 413*, 414*, 429.2, 746.85, V45.82 | I20*, I21*, I22*, 123*. I24*, I25*, Q24.5, Z95.5, Z98.61 |
| CHF | 402*, 404*, 428* | I09.81, I11.0, I11.9, I13*, I50* |
| PVD: Peripheral vascular disease | 443.0, 443.1, 443.82, 443.89, 443.9 | G64, I73.00, I73.01, I73.1, I73.81, I73.89, I73.9, I79.8 |
| Connective tissue disease | 710*, 711*, 712*, 713*, 714*, 715*, 716* | M30*-M36* |
| Ulcer disease | 531*, 532*, 533*, 534* | K25*, K26*, K27*, K28*, |
| GERD | 530.11, 530.81 | K21* |
| Liver disease | 570*, 571*, 572*, 573*, | K70*-K77* |
| Familial Dysautonomia (Riley-Day) | 742.8 (not specific: Other specified congenital anomalies of nervous system) | G90.1 |
| Asthma | 493* except COPD codes 493.20, 493.21, 493.22, | J45* |
| Allergic rhinitis | 477* | J30* |
| Atopic Dermatitis | 691.8 | L20* |
| Urticaria | 708* | L50* |
| Ulcerative colitis | 556.9 | K51* |
| Crohn’s disease (regional enteritis) | 555* | K50* |
| Anxiety | 300* | F40*, F41* |
| Depression | 296*, 311 | F31*, F32*, F33* |
| Dementia | 290*- 299*, 331*, 797 | F00*, F01*, F02*, F03*, F04*, F05*, F06* |
| Cognitive impairment | 310.9, 331.83, 799.52, 799.59, 780.99 | G31.84, ‎R41.8*, ‎R41.3, ‎R41.841, ‎R41.9, I69* |
| Migraine | 346* | G43* |
| Osteoporosis or osteopenia, osteomalacia | 733*, 268.2 | M80*, M81*, M83* |
| Arthritis (Osteo) | 715* | M15*-M19* |
| Anaphylaxis | 995*, 989.5, 999.41, 999.42, 999.49 | T78*, T80.51XA, T80.52XA, T80.59XA, T88.6XXA |
| Hepatosplenomegaly |  |  |
| Hepatomegaly | 789.1 | R16.0 |
| Splenomegaly | 789.2, 289.51 | R16.1, D73.2 |
| Lymphadenopathy | 785.6 | A18.2, R59* |
| Fracture | 800*- 829*, 733.1* | S02*, S12*, S22*, S32*, S42*, S52*, S62*, S72*, S82*, S92*, M80.0*, M80.8*, M84.3*, M84.4* |
| Syncope | 780.2 | R55* |
| Hypotension | 458.9 | I95* |
| IBS | 564.1 | K58* |
| IBD | 555*,556*,558.9 | K50*, K51*, K52.9 |

**Table E3. List of selected keywords/phrases for each symptom.**

| **Symptom** | **Keywords or phrases** |
| --- | --- |
| **Spots/Lesions/Hives** | Urticaria Pigmentosa, Erythematous brown macules, Yellow, red, brown or tan macules or papules or spots or lesions, Hyperpigmented macules or papules, Cutaneous Mastocytoma, Urticarial Eruption, Rash, Hives, Urticaria, Migratory lesions, Fixed lesions, Pruritic rash, Urticarial lesions, Maculopapular red-brown lesions, Generalized skin spots, Purpura, Pigmented lesions, Freckles, Lesions, Welts, Wheals, Dots |
| **Itching** | Mild generalized itching, Itching, Itchy, Itching sensation, Pruritus, Other pruritus, Chronic pruritus,  Episodic pruritus, Scratching, Bothered by Itching, Generalized pruritus, Intraoral pruritus, Itching of abdomen, Itching of ear, Itching of legs, Itching of soles, Itching papule, Labial itching, Larynx itching, Lip itching, Lip pruritus, Neck pruritus, Oral pruritus, Palmar pruritus, Periorbital pruritus, Pruritus aggravated, Pruritus allergic, Tongue pruritus, Usual Severity Itching, itch, Itchiness, Pruritic |
| **Flushing/Redness** | Flushing, Erythema, Flush(ed), Erythematous, Red rashes, Rosasceal flushing, Gross flushing, Generalized flushing, Flushing - episodic, Redness generalized, Redness of skin, Redness, Red papules, Red skin |
| **Burning** | Burning pain, Burning itch, Episodic burning pain, Burning skin, Burning, Intraoral burning, Burning of abdomen, Burning of legs, Burning of soles, Burning papule, Labial burning, Larynx burning, Lip burning, Oral burning, Palmar burning, Periorbital burning, Burning allergic, Burning pain, Burns, Burn, Skin on fire |
|  |  |
| **Swelling** | Angioedema, Edema, Swelling, Swelling of, Localized swelling, Inflammatory swelling, Intraoral swelling, Swelling of abdomen, Swelling of legs, Swelling of soles, Swelling papule, Labial swelling, Larynx swelling, Lip swelling, Oral swelling, Palmar swelling, Periorbital swelling, Swelling allergic, Swollen |
| **Abdominal pain/ Cramping** | Abdo pain, abdomen acute, Abdominal ache, Abdominal cramps, Abdominal pain, abdominal/abdomen colic, abdominal/abdomen cramp, abdominal/abdomen cramping, Abdominal/abdomen pain, Acute abdomen, Agony in abdo region, Agony in abdominal region, Belly pain, Colicky pain, Dull ache in tummy, Epigastric burning, Epigastric cramp, Epigastric discomfort, Epigastric Pain, Gastric pain, Intestinal pain, llq pain, lower quadrant pain, luq pain, pain abdominal, Pain in abdo region, pain in abdominal, Pain in abdominal region, Pain on abdominal wall movement, Lower abdomen pain, Parietal pain, Pelvic pain, rlq pain, ruq pain, Severe abdominal pain, Stomachache, stomach cramping, Stomach cramps, Stomach pain, Stomach aches, Upper quadrant pain, Visceral pain, Generalized abdominal pain, Pain under the rib cage, Gut ache, abd pain |
| **Epigastric or abdominal bloating** | Abdomen distended, Abdomen distended, Abdominal bloating, Abdominal distention, Abdominal distention epigastric, Abdominal gas, Acute dilatation of stomach, Bloating, Epigastric bloating, Epigastric distress, Epigastric fullness, Epigastric pressure, Epigastric swelling, Flatulence, Bloating and distension, Gas, Gas bloat, Inflamado, Inflammacion, Gas pain, abd pressure, Excess gassiness |
| **Diarrhea** | Diarrhea, Loose stools, Loosing stools, Watery stools, Loose bowel movements, Loosing bowel movements,  Water bowel movements, n/d, v/d, n/v/d, Liquid bowel movement |
| **Nausea or vomiting** | n\\v, n\v\d, nv, n-v, nvd, n-v-d, barf, barfed, barfing, emesis, nausea, puke, puked, puking, throw up, throwed up, throwing up, upchuck, upchucked, upchucking, vomit, vomited, vomiting, puking, puke, throwing up, vomit, vomiting, nausea, nauseated, nauseating, nauseous, N&V, N&V&D, N/V, N/V/D, NVD, vomitus |
| **Constipation** | Chronic constipation, Acute constipation, Constipation – functional, Perceived constipation, Slow transit constipation, Constipation, Constipated, Impacted stool |
| **Dizziness** | Vertigo, Postural dizziness, Exertional dizziness, Dizziness present, Multisensory dizziness, Dizziness intermittent, Dizziness chronic, Positional Dizziness, Episodic Dizziness, Recurrent dizziness, Dizzy, Dizziness |
| **Brain fog/Difficulty concentrating** | Brain fog, Trouble concentrating, Concentration problems, Have difficulty concentrating, Unable to concentrate, Impaired concentration, Poor concentration, Mental fuzziness, Mind fog, Foggy in my thought process, Not thinking clearly, Inability to focus, Inability to concentrate |
| **Headache** | Headache, Migraine, Hemicrania, Head pain, Head aching,  Head sore, Sinus pain, Phonophobia, Cephalgia, Temporal arteritis, Sore head, Aching head, Ached head, Sore on head, Pain on head, ache on head, Cranio-cervical instability |
| **Anxiety** | Anxiety, Anxiety and fear, Anxiety and panic, Anxiety Disorders, Anxiety hyperventilation, Anxiety neurosis, Chronic anxiety, Feeling of panic or panic feelings, Panic attacks due to general medical condition, Phobic anxiety disorder, Generalized Anxiety Disorder, Organic anxiety disorder, Panic Disorder, Performance anxiety, Phobia, Social or Social phobia, Separation Anxiety [Disorder], Social Anxiety [Disorder], Agoraphobia |
| **Depression** | Acute depression, Agitated depression, Atypical depressive disorder, Chronic depression, Depressed mood, Depression, Depression – recess, Depression – Bipolar, Depression – psychotic, Depressive - episode unspecified, Depressive Symptoms, Dysthymia, Masked depression, Melancholia, Mild depression, Mixed anxiety and depressive disorder, Recurrent depression, Recurrent major depressive episodes, Severe depression, Unipolar Depression |
| **Insomnia** | Acute insomnia, Can’t sleep, Cannot sleep, Chronic insomnia, Difficult getting to sleep, Difficult to sleep, Difficulty falling asleep, Difficulty sleeping, Difficulty staying asleep, Few hours of sleep, Insomnia, Loss of sleep, Lying awake for a long time at night, Not sleeping, Secondary insomnia, Sleep Initiation and Maintenance Disorders, Sleepless night, Sleeplessness, Trouble going back to sleep, Trouble sleeping, Troubled sleep, Wakes up during the night, Waking up during the night, Waking up too early, Hard time sleeping |
| **Bone pain** | Bony pain, Bone pain aggravated, Bone pain – periodic, Severe bone pain, Diffuse bone pain, Constant bone pain |
| **Joint pain** | Joint pain, Arthralgias |
| **Muscle pain** | Myalgia, Musculoskeletal Pain, Muscle pain – chronic, Severe muscle pain, Muscle pain |
| **Severe allergic reactions** | Anaphylactic Reaction, Anaphylactic Shock, Anaphylaxis, Mast cell-related anaphylaxis, Idiopathic anaphylaxis, Anaphylactic Episode, Severe anaphylaxis, Life-threatening anaphylaxis, Acute anaphylaxis, Recurrent anaphylaxis, Allergic anaphylactic episode, Systemic Anaphylaxis, Hymenoptera venom-triggered anaphylaxis, Anaphylactoid Episodes, Localized anaphylaxis, Cutaneous anaphylaxis, Food anaphylaxis, Severe allergic reaction |
| **Fatigue** | Abnormal fatigue, Asthenia, Asthenic, Chronic fatigue, Complaining of "tired all the time", Decreased energy,  Easy fatigue, Exhausted, Exhaustion, Fatigability, Fatigue, Fatigue Interferes with Mood, Fatigue – intermittent, Fatigue: Disruptive Effects, Feel Fatigue, Feeling tired, Generalized fatigue, Knackered, Lack of energy, Lacking energy, Lethargic, Lethargy, Loss of energy, TATT, Tired, Tired all the time, Tiredness, Weariness, Worn out, Enervated, Weary, Cancer-related fatigue, Low energy or lower energy, Decreased energy, Easily fatigue/fatigued, Decrease in energy, Not much energy, Energy level: low, Does not have energy, Not enough energy |
| **Syncope** | Syncope, Syncopal, Pass out, Passing out, Passed out, Fainting, Fainted, Black out, Blacked out, Lose consciousness,  Losing consciousness, Lost consciousness, Unconsciousness, Loss of consciousness |
|  |  |
| **Weight loss** | Body weight gone down, Decrease in body weight, Lose/losing weight, Losing weight, Loss of weight, Lost weight, Reduced body weight, Reduction in body weight, Wasting away, Weight + decrease/decreased/decreasing, Weight + loss, Weight + reduction, Weight decrease, Weight loses, Weight losing, Weight Loss, Weight Lost, wt loss |

**Table E4. Exclusion criteria for SM related symptoms.**

| **Symptom** | **Exclusion** |
| --- | --- |
|  | Inpatient notes/encounters for surgery and anesthesia (e.g. procedures, patient instructions, discharge instructions etc.) |
| All symptoms | Symptom only appeared in patient active problem list, except hospital active problem list  Symptom only appeared as medical history (i.e. not within last 30 days)  Symptom only appeared in instructions (e.g., patient instruction, medication instruction, discharge instruction)  Symptom only indicated by medication usage but prescribed as prn or as needed  Symptom only indicated by medication/treatment side effects, except severe allergic reactions |
| Swelling, redness, itching, burning | Symptom only indicated by trauma, surgery, injury, accident, bite/sting except for severe allergic reactions etc. |
| Swelling, redness, itching, burning | Symptom unrelated to skin (e.g. symptom in eye or internal organs such as burning of stomach, bladder etc.) |
| Spots, lesions or hives | Spots/lesions without color description (e.g. red, yellow, brown) |
| Itching | Itching of specific body parts (e.g. genitals, breast, hair/scalp) |
| Burning | Burning Mouth Sensation (BMS) |
| Nausea or vomiting | Hematemesis (vomiting of blood) |
| Anxiety | Symptom described as “stress”, “worry”, “anxious” |
| Insomnia | Symptom due to specific reasons, such as pain, UTI symptoms, etc. |
| Joint pain | Symptom only occurring in one joint |
| Severe allergic reactions | Allergic reactions that are not acute/immediate and severe |
| Fatigue | Physical fatigue |
| Syncope | Pre or near syncope |
| Weight loss | Intentional (in known), such as, weight loss program, regular exercises, on diet, etc. |

**Table E5. Rater Reliability Measures.**

| Symptoms | Accuracy | | | Gwet's AC1 | | | Kappa | | |
| --- | --- | --- | --- | --- | --- | --- | --- | --- | --- |
|  | Point Estimate | Conf Intervals | | Point Estimate | Conf Intervals | | Point Estimate | Conf Intervals | |
|  |  | 95% LL | 95% UL |  | 95% LL | 95% UL |  | 95% LL | 95% UL |
| **Cutaneous symptoms** |  |  |  |  |  |  |  |  |  |
| Spots, lesions or hives | 0.968 | 0.957 | 0.980 | 0.962 | 0.947 | 0.976 | 0.821 | 0.755 | 0.887 |
| Itching | 0.988 | 0.981 | 0.996 | 0.987 | 0.979 | 0.995 | 0.872 | 0.794 | 0.950 |
| Flushing or redness | 0.991 | 0.984 | 0.997 | 0.990 | 0.983 | 0.997 | 0.785 | 0.640 | 0.930 |
| Burning* | 0.997 | 0.993 | 1.000 | 0.997 | 0.993 | 1.000 |  |  |  |
| Swelling | 0.987 | 0.980 | 0.995 | 0.986 | 0.978 | 0.994 | 0.847 | 0.757 | 0.936 |
| **Gastrointestinal symptoms** |  |  |  |  |  |  |  |  |  |
| Abdominal pain or cramping | 0.997 | 0.993 | 1.000 | 0.996 | 0.992 | 1.000 | 0.901 | 0.791 | 1.000 |
| Epigastric or abdominal bloating | 1.000 | 1.000 | 1.000 | 1.000 | 1.000 | 1.000 | 1.000 | 1.000 | 1.000 |
| Diarrhea | 0.988 | 0.981 | 0.996 | 0.987 | 0.979 | 0.995 | 0.875 | 0.798 | 0.951 |
| Nausea or vomiting | 0.997 | 0.993 | 1.000 | 0.996 | 0.992 | 1.000 | 0.928 | 0.848 | 1.000 |
| Constipation | 0.997 | 0.993 | 1.000 | 0.996 | 0.992 | 1.000 | 0.901 | 0.791 | 1.000 |
| **Neuropsychiatric symptoms** |  |  |  |  |  |  |  |  |  |
| Dizziness | 0.995 | 0.991 | 1.000 | 0.995 | 0.991 | 1.000 | 0.748 | 0.509 | 0.987 |
| Brain fog or difficulty concentrating* | 1.000 | 1.000 | 1.000 | 1.000 | 1.000 | 1.000 |  |  |  |
| Headache | 0.992 | 0.986 | 0.998 | 0.991 | 0.985 | 0.998 | 0.894 | 0.817 | 0.972 |
| Anxiety | 0.998 | 0.994 | 1.000 | 0.998 | 0.994 | 1.000 | 0.888 | 0.733 | 1.000 |
| Depression | 0.997 | 0.993 | 1.000 | 0.996 | 0.992 | 1.000 | 0.921 | 0.833 | 1.000 |
| Insomnia | 0.999 | 0.997 | 1.000 | 0.999 | 0.997 | 1.000 | 0.909 | 0.730 | 1.000 |

| **Musculoskeletal symptoms** |  |  |  |  |  |  |  |  |  |
| --- | --- | --- | --- | --- | --- | --- | --- | --- | --- |
| Bone pain* | 0.998 | 0.994 | 1.000 | 0.998 | 0.994 | 1.000 |  |  |  |
| Joint pain | 0.997 | 0.993 | 1.000 | 0.996 | 0.992 | 1.000 | 0.901 | 0.791 | 1.000 |
| Muscle pain | 0.999 | 0.997 | 1.000 | 0.999 | 0.997 | 1.000 | 0.799 | 0.415 | 1.000 |
| **Severe allergic reactions*** | 0.999 | 0.997 | 1.000 | 0.999 | 0.997 | 1.000 |  |  |  |
| **Systemic symptoms** |  |  |  |  |  |  |  |  |  |
| Fatigue | 0.994 | 0.989 | 0.999 | 0.993 | 0.987 | 0.999 | 0.956 | 0.917 | 0.994 |
| Syncope* | 1.000 | 1.000 | 1.000 | 1.000 | 1.000 | 1.000 |  |  |  |
| Weight loss | 0.995 | 0.991 | 1.000 | 0.995 | 0.990 | 1.000 | 0.911 | 0.824 | 0.998 |

*Kappa statistics not available when either one rater scores every subject the same or two raters assign every subject the same rating.

**Table E6. Demographics and lifestyle characteristics of SM, ISM/SSM and Advanced SM patients, N (%).**

| **Patient demographics and lifestyle characteristics** | **SM (N=75)** | **Non-AdvSM (n=55)** | **AdvSM (n=15)** |
| --- | --- | --- | --- |
| Age |  |  |  |
| Mean (SD) | 58.7 (13.7) | 56.6 (12.8) | 66.6 (14.8) |
| Median (IQR) | 58 (47 - 68) | 57 (46 - 65) | 67 (58 - 80) |
| Range | 33 - 88 | 33 - 85 | 39 - 88 |
| Sex |  |  |  |
| Male | 41 (54.7) | 29 (49.2) | 12 (75.0) |
| Female | 34 (45.3) | 30 (50.8) | 4 (25.0) |
| Race/ethnicity |  |  |  |
| Non-Hispanic White | 41 (54.7) | 35 (59.3) | 6 (37.5) |
| Hispanic | 23 (30.7) | 16 (27.1) | 7 (43.8) |
| Black | 6 (8.0) | 3 (5.1) | 3 (18.8) |
| Asian/Pacific Islander | 4 (5.3) | 4 (6.8) | 0 (0.0) |
| Multiple/Other/Unknown | 1 (1.3) | 1 (1.7) | 0 (0.0) |
| Length of health plan enrollment in years, mean (SD) | 15.9 (15.5) | 14.0 (14.4) | 22.8 (18.0) |
| BMI |  |  |  |
| Mean (SD) | 28.3 (5.5) | 29.0 (5.9) | 25.5 (2.7) |
| Unknown | 2 (2.7) | 2 (3.4) | 0 (0.0) |
| Known | 73 (97.3) | 57 (96.6) | 16 (100) |
| Underweight/Normal | 23 (31.5) | 15 (26.4) | 8 (50.0) |
| Overweight | 28 (38.4) | 21 (36.8) | 7 (43.7) |
| Obese | 22 (30.1) | 21 (36.8) | 1 (6.3) |
| Exercise minute per week |  |  |  |
| Unknown | 17 (22.7) | 14 (23.7) | 3 (18.8) |
| Known | 58 (77.3) | 45 (76.3) | 13 (81.2) |
| Did not exercise | 18 (31.0) | 15 (33.3) | 3 (23.1) |
| Did exercise | 40 (69.0) | 30 (60.7) | 10 (76.9) |
| Minutes/wk, mean(SD) | 147.2 (157.2) | 154.7 (168.7) | 121.5 (110.2) |
| Minutes/wk, median(IQR) | 90 (0 - 240) | 90 (0 - 280) | 120 (20 - 240) |
| Smoking status |  |  |  |
| Unknown | 6 (8.0) | 6 (10.2) | 0 (0.0) |
| Known | 69 (92.0) | 53 (89.8) | 16 (100) |
| Yes | 2 (2.9) | 2 (3.8) | 0 (0) |
| Never | 45 (65.2) | 37 (69.8) | 8 (50.0) |
| Quit | 22 (31.9) | 14 (26.4) | 8 (50.0) |
| Passive | 0 (0) | 0 (0) | 0 (0) |
| Insurance types (mutually inclusive) |  |  |  |
| Commercial | 44 (58.7) | 38 (64.4) | 6 (37.5) |
| Medi-Cal/other State programs | 6 (8.0) | 4 (6.8) | 2 (12.5) |
| Medicare | 25 (33.3) | 15 (25.4) | 10 (62.5) |
| Private pay | 19 (25.3) | 13 (22.0) | 6 (37.5) |

**Table E7. Patient comorbidities in one year before and one year after index date in SM, ISM/SSM and Advanced SM patients, N (%).**

| **Patient comorbidities** | **SM (N=75)** | | **Non-AdvSM (n=55)** | | **AdvSM (n=15)** | |
| --- | --- | --- | --- | --- | --- | --- |
|  | **1 yr before** | **1 yr after** | **1 yr before** | **1 yr after** | **1 yr before** | **1 yr after** |
| Diabetes | 16 (21.3) | 16 (21.3) | 11 (18.6) | 11 (18.6) | 5 (31.3) | 5 (31.3) |
| Hypertension | 26 (34.7) | 30 (40.0) | 16 (27.1) | 20 (33.9) | 10 (62.5) | 10 (62.5) |
| Malignancy | 6 (8.0) | 5 (6.7) | 3 (5.1) | 3 (5.1) | 3 (18.8) | 2 (12.5) |
| Hyperlipidemia | 28 (37.3) | 28 (37.3) | 21 (35.6) | 20 (33.9) | 7 (43.8) | 8 (50.0) |
| Chronic Obstructive Pulmonary Disease | 3 (4.0) | 2 (2.7) | 3 (5.1) | 2 (3.4) | 0 (0) | 0 (0) |
| Cardiovascular Disease | 8 (10.7) | 11 (14.7) | 4 (6.8) | 6 (10.2) | 4 (25.0) | 5 (31.3) |
| Coronary Artery Disease | 5 (6.7) | 5 (6.7) | 4 (6.8) | 4 (6.8) | 1 (6.3) | 1 (6.3) |
| Myocardial Infarction | 1 (1.3) | 1 (1.3) | 1 (1.7) | 1 (1.7) | 0 (0) | 0 (0) |
| Coronary Heart Failure | 2 (2.7) | 6 (8.0) | 0 (0) | 2 (3.4) | 2 (12.5) | 4 (25.0) |
| Peripheral Vascular Disease | 1 (1.3) | 2 (2.7) | 0 (0) | 1 (1.7) | 1 (6.3) | 1 (6.3) |
| Connective tissue disease | 9 (12.0) | 7 (9.3) | 6 (10.2) | 4 (6.8) | 3 (18.8) | 3 (18.8) |
| Ulcer disease | 0 (0) | 1 (1.3) | 0 (0) | 0 (0) | 0 (0) | 1 (6.3) |
| Urticaria | 1 (1.3) | 4 (5.3) | 1 (1.7) | 4 (6.8) | 0 (0) | 0 (0) |
| Liver disease | 7 (9.3) | 8 (10.7) | 4 (6.8) | 4 (6.8) | 3 (18.8) | 4 (25.0) |
| Dysautonomia | 0 (0) | 0 (0) | 0 (0) | 0 (0) | 0 (0) | 0 (0) |
| IBS | 3 (4.0) | 5 (6.7) | 2 (3.4) | 4 (6.8) | 1 (6.3) | 1 (6.3) |
| IBD | 2 (2.7) | 6 (8.0) | 1 (1.7) | 1 (1.7) | 1 (6.3) | 5 (31.3) |
| Asthma | 8 (10.7) | 8 (10.7) | 8 (13.6) | 8 (13.6) | 0 (0) | 0 (0) |
| Allergic Rhinitis | 2 (2.7) | 5 (6.7) | 2 (3.4) | 5 (8.5) | 0 (0) | 0 (0) |
| Atopic Dermatitis | 0 (0) | 0 (0) | 0 (0) | 0 (0) | 0 (0) | 0 (0) |
| Anxiety | 15 (20.0) | 13 (17.3) | 13 (22.0) | 11 (18.6) | 2 (12.5) | 2 (12.5) |
| Depression | 11 (14.7) | 14 (18.7) | 10 (16.9) | 12 (20.3) | 1 (6.3) | 2 (12.5) |
| Dementia | 2 (2.7) | 4 (5.3) | 1 (1.7) | 3 (5.1) | 1 (6.3) | 1 (6.3) |
| Migraine | 2 (2.7) | 7 (9.3) | 2 (3.4) | 7 (11.9) | 0 (0) | 0 (0) |
| Cognitive impairment | 1 (1.3) | 1 (1.3) | 1 (1.7) | 1 (1.7) | 0 (0) | 0 (0) |
| Osteoporosis or osteopenia | 10 (13.3) | 18 (24.0) | 10 (16.9) | 14 (23.7) | 0 (0) | 4 (25.0) |
| Osteoarthritis | 15 (20.0) | 11 (14.7) | 11 (18.6) | 8 (13.6) | 4 (25.0) | 3 (18.8) |
| Fracture | 8 (10.7) | 8 (10.7) | 7 (11.9) | 7 (11.9) | 1 (6.3) | 1 (6.3) |
| Anaphylaxis | 8 (10.7) | 7 (9.3) | 7 (11.9) | 5 (8.5) | 1 (6.3) | 2 (12.5) |
| Hepatosplenomegaly |  |  |  |  |  |  |
| Hepatomegaly | 2 (2.7) | 0 (0) | 1 (1.7) | 0 (0) | 1 (6.3) | 0 (0) |
| Splenomegaly | 4 (5.3) | 4 (5.3) | 0 (0) | 0 (0) | 4 (25.0) | 4 (25.0) |
| Lymphadenopathy | 3 (4.0) | 2 (2.7) | 1 (1.7) | 0 (0) | 2 (12.5) | 2 (12.5) |
| GERD | 11 (14.7) | 11 (14.7) | 9 (15.3) | 8 (13.6) | 2 (12.5) | 3 (18.8) |
| Hypotension | 0 (0) | 2 (2.7) | 0 (0) | 0 (0) | 0 (0) | 2 (12.5) |

See ICD-9 and ICD-10 codes in eTable 1.

**Table E8. Patient lab characteristics in one year before and one year after index date in SM, ISM/SSM and Advanced SM patients, N (%).**

| **Lab characteristics** | **SM (N=75)** | | | | **Non-AdvSM (n=55)** | | | | **AdvSM (n=15)** | | | |
| --- | --- | --- | --- | --- | --- | --- | --- | --- | --- | --- | --- | --- |
|  | **1 yr before** | | **1 yr after** | | **1 yr before** | | **1 yr after** | | **1 yr before** | | **1 yr after** | |
|  | N | Pos (%) | N | Pos (%) | N | Pos (%) | N | Pos (%) | N | Pos (%) | N | Pos (%) |
| Anemia (Hb < 10 g/dL): Min | 69 | 6 (8.7) | 70 | 10 (14.3) | 53 | 1 (1.9) | 54 | 3 (5.6) | 16 | 5 (31.3) | 16 | 7 (43.8) |
| Leukopenia <3.5 x 10^9^/L: Min | 69 | 9 (13) | 69 | 10 (14.5) | 53 | 1 (1.9) | 54 | 2 (3.7) | 16 | 8 (50.0) | 15 | 8 (53.3) |
| Platelets < 100 x 10^9^/L: Min | 69 | 5 (7.2) | 69 | 9 (13.0) | 53 | 0 (0) | 54 | 1 (1.9) | 16 | 5 (31.3) | 15 | 8 (53.3) |
| Eosinophilia (>0.5 x 10^9^ /L): Max | 62 | 4 (6.5) | 64 | 5 (7.8) | 47 | 1 (2.1) | 49 | 3 (6.1) | 15 | 3 (20.0) | 15 | 2 (13.3) |
| Monocytosis (>1.0 x 10^9^/L): Max | 63 | 11 (17.5) | 64 | 12 (18.8) | 47 | 5 (10.6) | 49 | 5 (10.2) | 16 | 6 (37.5) | 15 | 7 (46.7) |
| Serum tryptase >20 ug/L: Max | 52 | 41 (78.8) | 59 | 45 (76.3) | 42 | 33 (78.6) | 47 | 34 (72.3) | 10 | 8 (80.0) | 12 | 11 (91.7) |
| Serum tryptase >200 ug/L: Max | 52 | 2 (3.8) | 59 | 6 (10.2) | 42 | 2 (4.8) | 47 | 5 (10.6) | 10 | 0 (0) | 12 | 1 (8.3) |
| Serum LDH >240 IU/L: Max | 19 | 0 (0) | 27 | 2 (7.4) | 9 | 0 (0) | 20 | 2 (10.0) | 10 | 0 (0) | 7 | 0 (0) |
| Serum alkaline phosphatase (SAP) >140 IU/L: Max | 54 | 5 (9.3) | 60 | 10 (16.7) | 42 | 3 (7.1) | 46 | 2 (4.3) | 12 | 2 (16.7) | 14 | 8 (57.1) |
| β2-Microglobulin >2.5 mg/L: Max | 2 | 1 (50.0) | 7 | 1 (14.3) | 1 | 0 (0) | 6 | 0 (0) | 1 | 1 (100) | 1 | 1 (100) |
| Serum cholesterol <120 mg/dL: Min | 55 | 10 (18.2) | 36 | 9 (25.0) | 44 | 5 (11.4) | 28 | 4 (14.3) | 11 | 5 (45.5) | 8 | 5 (62.5) |
| Serum vitamin B12 >1500 pg/mL: Max | 19 | 1 (5.3) | 16 | 1 (6.3) | 12 | 0 (0) | 13 | 1 (7.7) | 7 | 1 (14.3) | 3 | 0 (0) |
| Serum ferritin >200 ng/mL: Max | 14 | 8 (57.1) | 18 | 5 (27.8) | 6 | 2 (33.3) | 12 | 1 (8.3) | 8 | 6 (75.0) | 6 | 4 (66.7) |
| Serum ferritin <10 ng/mL: Min | 14 | 0 (0) | 18 | 0 (0) | 6 | 0 (0) | 12 | 0 (0) | 8 | 0 (0) | 6 | 0 (0) |
| Alanine transaminase (ALT) > 2 x upper limit: Max | 66 | 1 (1.5) | 65 | 1 (1.5) | 52 | 1 (1.9) | 51 | 0 (0) | 14 | 0 (0) | 14 | 1 (7.1) |
| Aspartate aminotransferase (AST) > 2 x upper limit: Max | 34 | 1 (2.9) | 33 | 1 (3.0) | 23 | 0 (0) | 24 | 0 (0) | 11 | 1 (9.1) | 9 | 1 (11.1) |
| C-reactive protein (CRP) >=7.5 mg/L: Max | 5 | 1 (20.0) | 0 | 0 (0) | 0 | 0 (0) | 0 | 0 (0) | 2 | 1 (50.0) | 0 | 0 (0) |
| Erythrocyte sedimentation rate (ESR) >=15 mm/hr: Max | 16 | 5 (31.3) | 12 | 3 (25.0) | 0 | 2 (0) | 8 | 1 (12.5) | 6 | 3 (50.0) | 4 | 2 (50.0) |

Min: the minimum value within each time window; Max: the maximum value within each time window.

**Table E9. Medication dispensing or administration in one year before and one year after index date in SM, ISM/SSM and Advanced SM patients, N (%).**

| **Dispensed medication** | **SM (N=75)** | | **Non-AdvSM (n=55)** | | **AdvSM (n=15)** | |
| --- | --- | --- | --- | --- | --- | --- |
|  | 1 yr before | 1 yr after | 1 yr before | 1 yr after | 1 yr before | 1 yr after |
| EPIPEN auto-injector | 17 (22.7) | 27 (36.0) | 15 (25.4) | 23 (39.0) | 2 (12.5) | 4 (25.0) |
| Epinephrine injection | 1 (1.3) | 4 (5.3) | 1 (1.7) | 3 (5.1) | 0 (0) | 1 (6.3) |
| Systemic steroids | 16 (21.3) | 22 (29.3) | 14 (23.7) | 16 (27.1) | 2 (12.5) | 6 (37.5) |
| Inhaled corticosteroid | 5 (6.7) | 6 (8.0) | 5 (8.5) | 6 (10.2) | 0 (0) | 0 (0) |
| H1 antihistamines | 16 (21.3) | 19 (25.3) | 14 (23.7) | 15 (25.4) | 2 (12.5) | 4 (25.0) |
| H2 antihistamines | 17 (22.7) | 26 (34.7) | 15 (25.4) | 23 (39.0) | 2 (12.5) | 3 (18.8) |
| Leukotriene receptor antagonists (LTRA) and 5-LO inhibitors | 4 (5.3) | 7 (9.3) | 4 (6.8) | 7 (11.9) | 0 (0) | 0 (0) |
| Cromolyn sodium | 5 (6.7) | 11 (14.7) | 4 (6.8) | 11 (18.6) | 1 (6.3) | 0 (0) |
| Chemo or targeted agents for SM | 0 (0) | 14 (18.7) | 0 (0) | 5 (8.5) | 0 (0) | 9 (56.3) |
| Osteoporosis drug | 6 (8.0) | 8 (10.7) | 5 (8.5) | 6 (10.2) | 1 (6.3) | 2 (12.5) |
| Biologics | 0 (0) | 0 (0) | 0 (0) | 0 (0) | 0 (0) | 0 (0) |
| Proton-pump inhibitor (PPI) | 13 (17.3) | 15 (20.0) | 10 (16.9) | 10 (16.9) | 3 (18.8) | 5 (31.3) |
| Disease modifying anti-rheumatic drugs (cyclosporine, methotrexate) | 0 (0) | 0 (0) | 0 (0) | 0 (0) | 0 (0) | 0 (0) |

**Table E10. Symptoms in one year before and one year after index date in SM, CSU and non-SM/non-CSU patients, N (%).**

| **Symptoms** | SM (N=70) | | CSU (N=140) | | Non-SM/non-CSU (N=140) | |
| --- | --- | --- | --- | --- | --- | --- |
|  | 1 yr before | 1 yr after | 1 yr before | 1 yr after | 1 yr before | 1 yr after |
| **Cutaneous symptoms** | 52 (74.3) | 63 (90.0) | 113 (80.7) | 136 (97.1) | 34 (24.3) | 51 (36.4) |
| Spots, lesions or hives | 48 (68.6) | 58 (82.9) | 108 (77.1) | 136 (97.1) | 20 (14.3) | 36 (25.7) |
| Itching | 22 (31.4) | 37 (52.9) | 92 (65.7) | 124 (88.6) | 11 (7.9) | 12 (8.6) |
| Flushing or redness | 19 (27.1) | 25 (35.7) | 54 (38.6) | 71 (50.7) | 7 (5.0) | 11 (7.9) |
| Burning | 3 (4.3) | 3 (4.3) | 9 (6.4) | 11 (7.9) | 1 (0.7) | 3 (2.1) |
| Swelling | 13 (18.6) | 23 (32.9) | 63 (45.0) | 89 (63.6) | 19 (13.6) | 25 (17.9) |
| **Gastrointestinal symptoms** | 35 (50.0) | 45 (64.3) | 51 (36.4) | 52 (37.1) | 29 (20.7) | 41 (29.3) |
| Abdominal pain or cramping | 15 (21.4) | 25 (35.7) | 28 (20.0) | 29 (20.7) | 18 (12.9) | 23 (16.4) |
| Epigastric or abdominal bloating | 7 (10.0) | 13 (18.6) | 6 (4.3) | 13 (9.3) | 6 (4.3) | 6 (4.3) |
| Diarrhea | 16 (22.9) | 32 (45.7) | 14 (10.0) | 21 (15.0) | 12 (8.6) | 15 (10.7) |
| Nausea or vomiting | 18 (25.7) | 25 (35.7) | 30 (21.4) | 25 (17.9) | 14 (10.0) | 23 (16.4) |
| Constipation | 15 (21.4) | 16 (22.9) | 11 (7.9) | 11 (7.9) | 6 (4.3) | 14 (10.0) |
| **Neuropsychiatric symptoms** | 33 (47.1) | 40 (57.1) | 70 (50.0) | 72 (51.4) | 40 (28.6) | 53 (37.9) |
| Dizziness | 7 (10.0) | 17 (24.3) | 29 (20.7) | 24 (17.1) | 14 (10.0) | 18 (12.9) |
| Brain fog or difficulty concentrating | 3 (4.3) | 8 (11.4) | 3 (2.1) | 5 (3.6) | 1 (0.7) | 6 (4.3) |
| Headache | 13 (18.6) | 26 (37.1) | 37 (26.4) | 43 (30.7) | 18 (12.9) | 28 (20.0) |
| Anxiety | 21 (30.0) | 17 (24.3) | 25 (17.9) | 28 (20.0) | 18 (12.9) | 18 (12.9) |
| Depression | 9 (12.9) | 14 (20.0) | 21 (15.0) | 26 (18.6) | 10 (7.1) | 14 (10.0) |
| Insomnia | 9 (12.9) | 11 (15.7) | 15 (10.7) | 17 (12.1) | 10 (7.1) | 11 (7.9) |
| **Musculoskeletal symptoms** | 12 (17.1) | 20 (28.6) | 24 (17.1) | 38 (27.1) | 27 (19.3) | 31 (22.1) |
| Bone pain | 1 (1.4) | 4 (5.7) | 1 (0.7) | 1 (0.7) | 1 (0.7) | 0 (0) |
| Joint pain | 7 (10.0) | 14 (20.0) | 17 (12.1) | 27 (19.3) | 14 (10.0) | 20 (14.3) |
| Muscle pain | 6 (8.6) | 8 (11.4) | 14 (10.0) | 20 (14.3) | 13 (9.3) | 16 (11.4) |
| **Severe allergic reactions*** | 4 (5.7) | 6 (8.6) | 2 (1.4) | 2 (1.4) | 0 (0) | 0 (0) |
| **Systemic symptoms** | 26 (37.1) | 31 (44.3) | 37 (26.4) | 46 (32.9) | 27 (19.3) | 43 (30.7) |
| Fatigue | 20 (28.6) | 28 (40.0) | 32 (22.9) | 41 (29.3) | 26 (18.6) | 41 (29.3) |
| Syncope | 3 (4.3) | 3 (4.3) | 3 (2.1) | 8 (5.7) | 0 (0) | 2 (1.4) |
| Weight loss | 10 (14.3) | 15 (21.4) | 8 (5.7) | 8 (5.7) | 3 (2.1) | 5 (3.6) |

*Including anaphylaxis.

**Table E11. Symptoms in one year before and one year after index date in SM, ISM/SSM and Advanced SM patients****, N (%).**

| **Symptoms** | **SM (N=70)** | | **Non-AdvSM (n=55)** | | **AdvSM (n=15)** | |
| --- | --- | --- | --- | --- | --- | --- |
|  | **1 yr before** | **1 yr after** | **1 yr before** | **1 yr after** | **1 yr before** | **1 yr after** |
| **Cutaneous symptoms** | 52 (74.3) | 63 (90.0) | 43 (78.2) | 51 (92.7) | 9 (60.0) | 12 (80.0) |
| Spots, lesions or hives | 48 (68.6) | 58 (82.9) | 41 (74.5) | 48 (87.3) | 7 (46.7) | 10 (66.7) |
| Itching | 22 (31.4) | 37 (52.9) | 20 (36.4) | 32 (58.2) | 2 (13.3) | 5 (33.3) |
| Flushing or redness | 19 (27.1) | 25 (35.7) | 17 (30.9) | 21 (38.2) | 2 (13.3) | 4 (26.7) |
| Burning | 3 (4.3) | 3 (4.3) | 2 (3.6) | 2 (3.6) | 1 (6.7) | 1 (6.7) |
| Swelling | 13 (18.6) | 23 (32.9) | 10 (18.2) | 14 (25.5) | 3 (20.0) | 9 (60.0) |
| **Gastrointestinal symptoms** | 35 (50.0) | 45 (64.3) | 28 (50.9) | 31 (56.4) | 7 (46.7) | 14 (93.3) |
| Abdominal pain or cramping | 15 (21.4) | 25 (35.7) | 9 (16.4) | 18 (32.7) | 6 (40.0) | 7 (46.7) |
| Epigastric or abdominal bloating | 7 (10.0) | 13 (18.6) | 4 (7.3) | 5 (9.1) | 3 (20.0) | 8 (53.3) |
| Diarrhea | 16 (22.9) | 32 (45.7) | 14 (25.5) | 20 (36.4) | 2 (13.3) | 12 (80.0) |
| Nausea or vomiting | 18 (25.7) | 25 (35.7) | 15 (27.3) | 16 (29.1) | 3 (20.0) | 9 (60.0) |
| Constipation | 15 (21.4) | 16 (22.9) | 12 (21.8) | 10 (18.2) | 3 (20.0) | 6 (40.0) |
| **Neuropsychiatric symptoms** | 33 (47.1) | 40 (57.1) | 29 (52.7) | 31 (56.4) | 4 (26.7) | 9 (60.0) |
| Dizziness | 7 (10.0) | 17 (24.3) | 7 (12.7) | 13 (23.6) | 0 (0) | 4 (26.7) |
| Brain fog or difficulty concentrating | 3 (4.3) | 8 (11.4) | 3 (5.5) | 8 (14.5) | 0 (0) | 0 (0) |
| Headache | 13 (18.6) | 26 (37.1) | 11 (20.0) | 21 (38.2) | 2 (13.3) | 5 (33.3) |
| Anxiety | 21 (30.0) | 17 (24.3) | 20 (36.4) | 13 (23.6) | 1 (6.7) | 4 (26.7) |
| Depression | 9 (12.9) | 14 (20.0) | 9 (16.4) | 12 (21.8) | 0 (0) | 2 (13.3) |
| Insomnia | 9 (12.9) | 11 (15.7) | 7 (12.7) | 8 (14.5) | 2 (13.3) | 3 (20.0) |
| **Musculoskeletal symptoms** | 12 (17.1) | 20 (28.6) | 8 (14.5) | 13 (23.6) | 4 (26.7) | 7 (46.7) |
| Bone pain | 1 (1.4) | 4 (5.7) | 1 (1.8) | 2 (3.6) | 0 (0) | 2 (13.3) |
| Joint pain | 7 (10.0) | 14 (20.0) | 4 (7.3) | 9 (16.4) | 3 (20.0) | 5 (33.3) |
| Muscle pain | 6 (8.6) | 8 (11.4) | 4 (7.3) | 6 (10.9) | 2 (13.3) | 2 (13.3) |
| **Severe allergic reactions*** | 4 (5.7) | 6 (8.6) | 4 (7.3) | 6 (10.9) | 0 (0) | 0 (0) |
| **Systemic symptoms** | 26 (37.1) | 31 (44.3) | 19 (34.5) | 19 (34.5) | 7 (46.7) | 12 (80.0) |
| Fatigue | 20 (28.6) | 28 (40.0) | 15 (27.3) | 18 (32.7) | 5 (33.3) | 10 (66.7) |
| Syncope | 3 (4.3) | 3 (4.3) | 3 (5.5) | 2 (3.6) | 0 (0) | 1 (6.7) |
| Weight loss | 10 (14.3) | 15 (21.4) | 6 (10.9) | 6 (10.9) | 4 (26.7) | 9 (60.0) |

*Including anaphylaxis.
